# Novel Technologies for Exosome and Exosome-like Nanovesicle Procurement and Enhancement

**DOI:** 10.3390/biomedicines11051487

**Published:** 2023-05-19

**Authors:** Andrés Martínez-Santillán, José González-Valdez

**Affiliations:** School of Engineering and Science, Tecnologico de Monterrey, Av. Eugenio Garza Sada 2501, Monterrey 64849, Mexico

**Keywords:** exosome, exosome-like nanovesicles, alternative extracellular vesicle sources, exosome functionalization

## Abstract

Exosomes are extracellular nanovesicles commonly produced by mammalian cells that in recent years have risen as a novel strategy for drug delivery systems and cancer therapy because of their innate specificity and high bioavailability. However, there are limitations that undermine their potential. Among them is the lack of mass production capacity with the current available sources and the failure to reach the intended therapeutic effect because of their insufficient uptake or their rapid clearance once administered. This review aims to show the current advances in overcoming these limitations by presenting, firstly, reported strategies to improve exosome and exosome-like nanovesicle extraction from possible novel eukaryotic sources, including animals, plants, and protozoa; and secondly, alternative modification methods that functionalize exosomes by conferring them higher targeting capacity and protection from organism defenses, which results in an increase in the attachment of ligands and cellular uptake of inorganic materials. However, even when these strategies might address some of the obstacles in their procurement and therapeutic use, there are still several aspects that need to be addressed, so several perspectives of the matter are also presented and analyzed throughout this work.

## 1. Introduction

**Figure 1:**
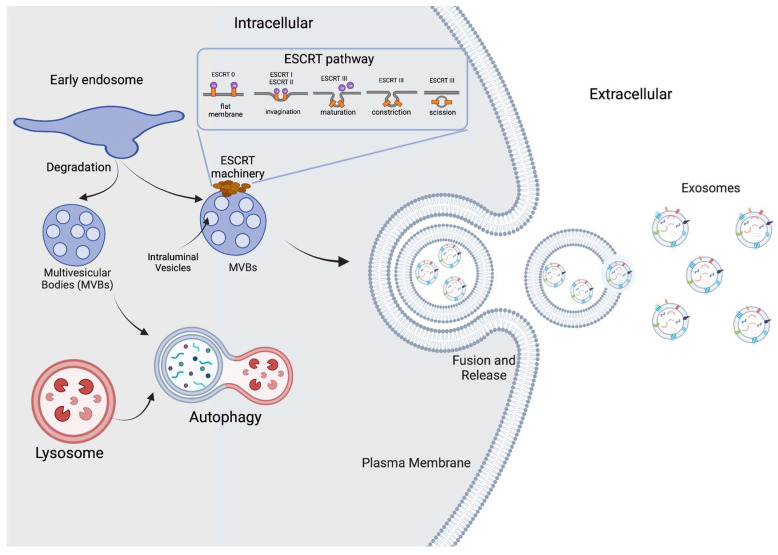
Exosome biogenesis. Small extracellular vesicles are formed inside early endosomes regulated by the endosomal sorting complexes required for transport (ESCRT). Invagination of late endosomal membranes results in the formation of intraluminal vesicles (ILVs) inside multivesicular bodies (MVBs). Finally, multivesicular bodies fuse with the plasma membrane and then release the exosomes to the exterior. Some MVBs follow a different pathway to degradation with the help of lysosomes.

Small extracellular vesicles, commonly known as exosomes, are specialized nanovesicles produced in mammalian cells, ranging in size from 30 to 150 nm, that derive from the fusion of an endocytic compartment, called a multivesicular body, with the cell membrane. This fusion results in a luminal vesicle with a lipid layer and embedded proteins [1]. Figure 1 shows a representation of exosomal biogenesis. There are various pathways for exosome biogenesis; the most analyzed is the one that uses endosomal sorting complexes required for transport (ESCRT). Designated with numbers from 0 to 3, these complexes carry different steps in exosome formation and can recognize ubiquitylated compounds due to their protein composition [2]. ESCRT 0 starts the process of recognizing ubiquitylated compounds and adding up proteins that will help to group the cargo. Both ESCRT-1 and ESCRT-2 help ESCRT-0 to establish a sorting domain that possesses a high preference for ubiquitylated cargo in the location where intraluminal vesicles will be formed. Finally, ESCRT-3 is recruited and modifies the cell membrane, so invagination is caused, and the vesicles are released [3]. The main known natural functions of the exosomal vesicles inside the organism are to dispose of unneeded cell materials and to provide cell-to-cell communication through the transmission of macromolecules [4]. Even when exosomes are common to mammalian cells, plant and bacterial cells also produce exosome analogues and other extracellular nanovesicles.

Exosome content depends on the type of cell from which they are secreted, but also includes a regulated sorting mechanism. In both the membrane and inside the nanovesicle, various proteins such as receptors, enzymes, and transcription factors can be found, as well as nucleic acids (mRNA, miRNA) and lipids. Some of these components are common across different types of exosomes, while others are reliant on the original tissue [5]. Some of the molecules found in the membranes and the lumen of these small extracellular vesicles play a role in cell-to-cell communication processes. To date, there are three known mechanisms for information delivery through exosomes: the interaction between exosomal proteins and target cell receptors; the fusion of the exosome with the cell membrane, which then deposits its contents into the cytosol; and finally, internalization, a process similar to phagocytosis where the exosome is invaginated into the cytosol [6]. Exosome-mediated information transfer is of great relevance since it has also been stated that exosomes play a role in viral infection through the hijacking of the internal cell machinery [7], and also in cancer metastases, as tumor-derived exosomes can remodel surrounding cells for the proliferation of malignant cells in tumor microenvironments [8].

Furthermore, because of their ability to cross biological barriers, natural functionality and high specificity, exosomes have been used in the development of clinical applications, such as targeted drug delivery systems, cancer therapy, and anti-inflammatory and regenerative therapies, just to mention some examples [9]. They also present utility as diagnostic biomarkers with the usage of fluorescent proteins. All these applications have been tested in both in vitro and in vivo trials [10]. In this context, Table 1 summarizes the different types of clinical applications that nanovesicles have shown promise in, as well as their source and some specific examples.

Even though there are many advantages to the use of exosomes, there are some limitations for their industrial development, production and application in new therapies. One of these limitations is the lack of process scalability. This problem originates from the commonly used extraction methods that either do not attain enough yield or cause the process to be too expensive and unprofitable in the end. Another problem is the incapacity of high quality and uniform collection due to the natural variation that exists within exosome sizes [20]. Lastly, exosomes present a rapid clearance by the organism that hampers the delivery of therapeutic molecules. This issue may originate from the administration route since, for example, exosomes have been observed to be cleared within the first two hours after injected applications or topic treatments to be ultimately accumulated in organs such as the liver and the gastrointestinal tract [21].

However, there have been some advancements to try to overcome these problems. Most of them are mainly focused on new extraction methods to ensure uniformity and higher production yields as well as modifying the surface of the exosome to improve its therapeutic characteristics [22]. This review seeks to present two different strategies to clear these limitations—one targeting possible new scalable sources from where to harvest exosomes, and the other one regarding their optimization as delivery systems via modification so that they reach the intended targets before being cleared by the organism.

## 2. Novel and Scalable Exosome Sources

**Figure 2:**
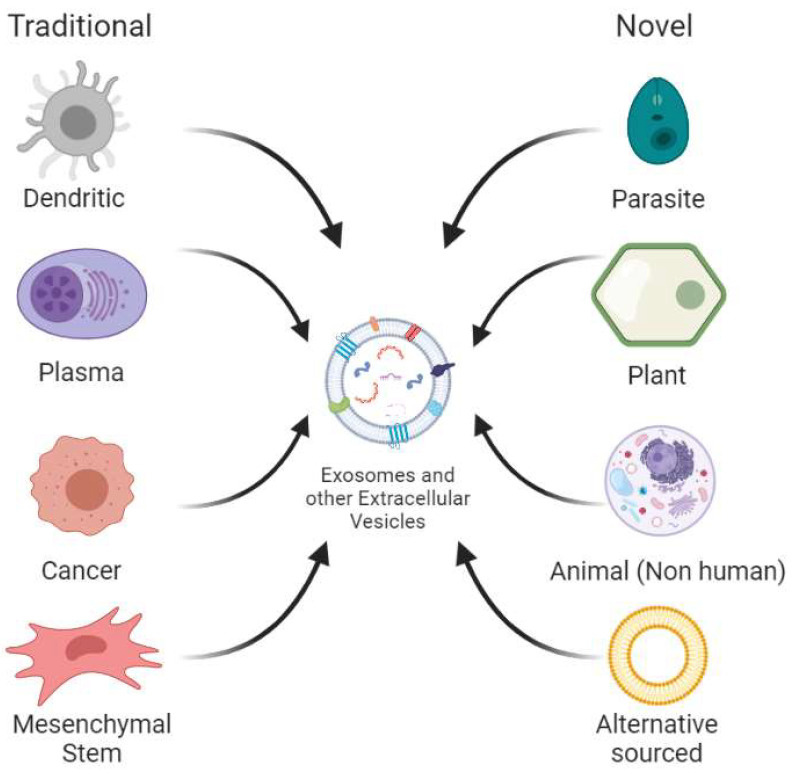
Traditional and novel sources for extracellular vesicle extraction. Traditional sources mainly refer to different cell types extracted from human fluids or produced via tissue culture. Novel sources include all other non-human animal sources, plants, parasites and those alternatively sourced, the latter referring to synthetically engineered extracellular vesicles.

Extracellular vesicles are commonly obtained from cell culture or mammalian fluids and present a wide array of characteristics depending on the cell from which they are obtained. An important example is in immune cells (e.g., dendritic cells, T cells, B cells, macrophages, and natural killer cells), as it has been found that exosomes help to regulate their action mechanisms. Exosomes from these sources have mainly been used to produce vaccines for certain diseases [23]. Traditional human exosome sources have proven to be inefficient in the production of these vesicles and most systems are usually unable to be scaled to supply the high demand needed for industrialized use [20]. Because of this reason, there has been an interest in the search for novel exosome sources by exploring other species in the animal kingdom—species of different kingdoms such as plants and parasitic prokaryotes—and even designing new systems from synthetic origin. Figure 2 presents a diagram that summarizes both the traditional and novel sources used to find new exosome and other extracellular nanovesicle sources, which will be explained in the following subsections.

### 2.1. Animal Sources

Exosomes can be found in bodily fluids such as blood and milk, the latter being the most well-suited for production using non-human animal sources such as yaks and cows. These animals can produce many liters of milk daily, opening the possibility for better exosome production yields compared to immortalized cell lines and blood. Moreover, new evidence suggests that bovine milk-based exosomes can carry information in the form of miRNAs that can be absorbed by human macrophages, affecting cell function. This indicates that therapeutic applications from this source are possible and can overcome the limitations found for other applications [24].

As is known, siRNAs are used in this context against certain diseases by silencing genes, but they are difficult to deliver successfully. Modifications to improve the delivery pathway have also been unsuccessful. This limitation, however, was overcome by using milk extracellular vesicles as vehicles to carry siRNAs. The exosomes were loaded with cargo to target specific genes such as: VEGF, EGFR, AKT, MAPK and KRAS. They were then enriched with folic acid to improve their therapeutic effects, showing an increased efficiency in the targeting and accumulation of nanovesicles in the tumor site. Anticancer properties—cellular uptake, gene silencing, antiproliferation and antitumor activity—were measured against lung tumor xenografts in in vitro and in vivo experiments in mice models. The xenografts showed that the tumor volume was reduced to less than half when compared to the control group. The antiproliferation assays, however, showed that the treatment did not affect the target cells [25].

Further studies have shown the possibility of using milk exosomes as carriers for pharmaceutical cargo to treat several diseases. In the study presented by Munagala et al., milk-derived exosomes were used for anticancer treatment against lung and breast human cancer cell lines. The extraction was performed by differential centrifugation, and then anticancer activity and cellular uptake were measured. These withaferin A-loaded exosomes presented a tumor growth inhibition twice as large as that obtained by using the anticancer agent without a carrier. In fact, the milk-derived exosomes showed a growth inhibition of 15–45% by themselves, depending on dose and cell line. They also presented anti-inflammatory effects without the need for carrying specialized drugs and improved function when coupled with anticancer agents [26].

One of the main advantages of using milk as a source for exosome production is that it appears that these particular type of vesicles present a high oral bioavailability as they can easily be absorbed in the gastrointestinal tract. This opens the possibility for the development of oral-based pharmaceuticals. In this regard, milk-derived exosomes have been labeled with DIR (1,1′-Dioctadecyl-3,3,3′,3′-Tetramethylindotricarbocyanine Iodide) and conjugated with the peptide ligand iRGD, a variant of Arginylglycylaspartic acid (RGD), which is known to target tumorigenic cells. In this study, the previously mentioned formulation was administered orally and compared to unaltered exosomes and liposomes to observe the uptake in various organs of cancer-ridden mice. iRGD exosomes showed the highest uptake by a large margin, up to four times compared to that of oral- and intravenous-administered exosomes, which shows that milk exosomes can be useful for administering intravenous drugs in an oral manner [27]. In another study, milk-derived exosomes were also used for the oral delivery of the anticancer drug paclitaxel, improving the overall efficacy of the drug as well as reducing the cytotoxicity that is associated with this type of treatment, opening a pathway for more safe therapies to fight cancer and other diseases [28].

The delivery of other active nutraceutical compounds such as anthocyanins and anthocyanidins has also been proposed using milk-derived exosomes as carriers. These compounds are found in different fruits and berries and present antioxidant and anticancer properties [29]. However, due to their lack of optimal pharmacokinetic characteristics traditional drug development opportunities have been scarce. In this sense, milk-derived exosomes were used to surpass these limitations. After loading, the antiproliferative activity was tested in five different cancer cell lines and antitumor and toxicity effects were studied in vivo using mice as test subjects. Results show that these milk-derived exosomes can cover the deficiencies that strategies that use bioactive compounds by themselves are present with. The antiproliferative assay showed a decrease in cell survival and was up to 60 times more effective than just using the nutraceutical actives alone. For the anti-inflammatory effects, the TNFα-induced NF-κB activity was evaluated. The results showed that low concentrations of anthocyanins and anthocyanidins (10 mg/kg) have little to no effect on suppressing the caused response; however, a smaller concentration (5 mg/kg) when coupled with exosomes (50 mg/kg) does, and the end result was around six times more effective [30].

Most of the studies are centered around bovine milk, however, milk-derived exosomes from other animals have also been explored. Examples of this include yak milk exosomes, which have been used for their anti-inflammatory properties. In a particular study conducted by Gao et al., yak milk-derived exosomes and their proteins were characterized and were observed to have a positive impact on intestinal epithelial cells when injured by lipopolysaccharides; this is caused by the activation of the PI3K/AKT/C3 pathway, which ultimately helps cell survivability [20]. Another example can be found in porcine models, as small extracellular vesicles from porcine milk were found to provide intestinal immunity as they promote the secretion of immunoglobulin A and also cause an increase in the expression of the polymeric immunoglobulin receptor; this effect was achieved in both mice and piglets [31].

Milk is not the only animal-based source for exosomes that has been studied for clinical or other purposes, though it may be the most scalable one. For instance, seminal plasma from boars was investigated and it was discovered to contain exosomes with the CD44 ligand. This evidence might be useful for determining that exosomes found in complex fluids carry different signaling molecules on the surface depending on their source, making it possible to fractionate them into different groups. More studies need to be carried out to better understand how these exosomes are produced and what applications they might have [32]. Another important feature to consider is that exosomal composition for each mammal is different, so it is necessary to determine if they can have the same type of applications or even if they are compatible with humans [33]. Another important factor to address is the content of the nanovesicles as evidence suggests that exosomes vary in proteome composition depending on the lactation stage—significant differences were found between the colostrum and mature milk-derived exosomes in a porcine model [34].

### 2.2. Plant Sources

Most of the studies regarding exosomes are targeted towards mammalian cells because it was initially thought that these were the only source for such vesicles. Recent studies, however, show the existence of similar exosome-like vesicles in plants that appear to carry a similar cell-to-cell communication function to animal organisms. However, there is still a lot of unknown information in that matter. Nonetheless, one of their apparent biological roles is in the defense against exogenous pathogenic organisms such as fungi, which relates these exosome-like vesicles with defense and immunity mechanisms and information transfer across cells [35].

Additional studies have related the presence of exosome-like vesicles in plants to responses against either biotic or abiotic stress. In this regard, apoplastic fluids from Arabidopsis were used to extract exosomes to then conduct further analyses. The proteomics revealed a heavy concentration of proteins related to stress responses. This shows that to optimize the production of plant-derived extracellular nanovesicles the organism must be under stressful conditions [36].

Other than function, it is also important to understand the composition of these plant-derived vesicles and compare them to the ones obtained from mammal sources, as extraction methods and functionalization procedures are dependent on their structure. These are different and usually require protocols that do not align with what is commonly used for mammalian sources. Separation by buoyant density, for example, is one of the methods that has shown some results—using differential centrifugation and sucrose/deuterium oxide ultracentrifugation, exosome-like vesicles were extracted from fruit juice and the protein yield was measured, resulting in 20 μg of protein in 1 mL of juice (clementine juice in this case). The authors note, however, that the overall yield will change depending on the starting material [37]. Please note that protein concentration is commonly used to indicate vesicle concentration, as the embedded proteins in the vesicle membrane are easy to measure spectrophotometrically [38].

It is also important to evaluate if these vesicles can interact or present any effect in mammal cells. Proving this opens the possibility for using them as a platform for clinical applications. For instance, Mu et al. tested the effects and the existence of interspecies communication between plant-sourced nanovesicles and mice. The vesicles were extracted from five different edible plants via centrifugation. Then, in vivo and in vitro experiments were carried out to observe the resistance to enzymatic digestion and then observe the uptake in macrophages and intestinal cells. It was found that these exosome-like vesicles had the capacity to induce the expression of genes for anti-inflammatory and antioxidant purposes in the animal subjects [39].

It is also known that many plant extracts are used in traditional medicine because of various beneficial properties. In this context, a study was carried out using wheat extract to determine if wheat-derived exosomes affected the compound’s known wound-healing capabilities. In vitro experiments were conducted to evaluate the cell viability and migration of certain molecules. The results showed an increase in the migration and proliferation of dermal cells. There was also no significant presence of apoptotic cells, and in general, the process did not affect the mechanisms inside the cell cycle, and the expression of collagen type I was also increased. Using these exosomes for their innate healing capabilities may be a plausible approach for clinical use in superficial wounds [40].

Furthermore, recent studies have shown the capacity that plant-derived extracellular vesicles present as drug delivery systems is comparable to their animal-based counterparts, with the added advantages of intrinsic natural therapeutic activity as well as a higher production and scalability potential [41]. However, more studies need to be performed to explore the limitations and the complete functionality and composition of plant exosomes before they can be used as optimal nanotherapeutics.

### 2.3. Parasitic Sources

Parasitic protozoa are other types of organisms that have been found to produce nanovesicles with similar characteristics to exosomes. The natural function of the vesicles is the same as with the other sources (i.e., intercellular communication and genetic material transfer), which can occur between host and parasite, or between parasites. The main goal in the former is to control the immune responses of the hosts, and in the latter, it is related with reproduction mechanisms to increase transmission [42]. It has been found that proteins from parasite-derived exosome-like molecules are related to general functionalities of the parasite’s development and survivability through its life cycle, while the miRNAs released help with the pathogenicity and interaction with host cells [43].

Parasites that are known to use exosome-like vesicles, and those that have been studied for this belong to the Leishmania genus. Exosome-like vesicles have been found to impact leishmaniasis progression through the incapacitation of host defenses and macrophage dysfunction. The mechanism of action includes a secretion of these vesicles within the parasite vector (in this case, the fly midgut), and then both the parasite and the vesicles are egested during the blood meal, causing an increase in inflammatory cytokines that help with pathogenicity [44]. Viruses such as Leishmania RNA Virus 1 have been detected to utilize parasite-based exosomes to protect themselves from external environmental conditions, which also confer them with a disguise coating so they can enter and proliferate in new cells while enhancing and modulating the detrimental effects and functions of mucocutaneous leishmaniasis [45].

Another example of a studied protozoa in this regard is the gastric parasite Heligmosomoides polygyrus, a nematode that is known to infect mice, capable of transferring miRNAs and Y RNAs to its host via exosome-like vesicles that function as protection for the RNAs to avoid degradation. These RNAs cause an inhibition in the activity of Type 2 innate responses and eosinophilia inside mice. To evaluate this effect, an in vivo test was conducted to observe the uptake of exosomes, and in vitro experiments were performed in mice epithelial cells. The study showed that both miRNAs and Y RNAs packed in exosome-like vesicles were secreted in detectable quantities. However, it is still unknown if these results are due to the environment manipulation by the parasite inside the host [46].

The potential applications for protozoan-based exosome-like vesicles rely on the creation of vaccines that take advantage of the pathways that parasites already use for natural infection, as well as the protection that comes with them [47]. Even though there are some possible impactful new insights and applications with these nanovesicles, it is hard to glimpse how they can be used for industrial production. However, future advancements may develop some therapies that can be more economically attractive than the alternatives used today, so it may be important to continue such studies to better understand the associated infection mechanisms.

### 2.4. Synthetic Alternatives

Manmade extracellular-like vesicles are another strategy that has been developed to sort production problems and to improve some inherent characteristics that natural exosomes present. An advantage regarding synthetic exosomes compared to their natural counterparts is that they can be customized with a controlled composition to better meet their functional and/or biological objective.

There are three main different strategies to create a synthetic exosome. The first category is the top-down approach, the second type is called a bottom-up strategy, and the final is by a biohybrid strategy; each of the three have many different methods for generating the artificial nanovesicles [48]. In the top-down strategy, artificial exosomes are made by disintegrating the parent cells into smaller parts, and the elements are dissembled to form these nanovesicles. Cells are force-passed through porous membranes using microfluidic devices and are disintegrated by sonication or nitrogen cavitation, to name some examples [49]. Bottom-up strategies consist of the creation of artificial exosomes by beginning with smaller molecules and then building larger and more-complex particles with desirable chemical and physical characteristics. This can be conducted by having liposomes as a base and adding certain compounds such as polymers and antibodies into their membranes [50]. The third and final biohybrid approach consists of merging synthetic nanoparticles with natural nanovesicles. There are various methods to achieve this, including freeze-thawing, natural incubation, PEG-mediated fusion and membrane extrusion [51].

An example of a bottom-up strategy in the creation of such vesicles is the exosome-mimetic nanosystem, which was designed for targeted cancer drug delivery using the ethanol injection method. These vesicles were created to deliver therapeutic oligonucleotides to combat against lung adenocarcinoma cells. The results were comparable to those that can be obtained with natural exosomes, with the advantages previously stated, plus size control, higher yield, and better adaptability for different applications [52].

Another instance of the creation of exosome mimics was developed by Yang et al. In this study, a top-down strategy was used. In this case, the mimics were generated by serial extrusion, using different sized filters. siRNAs of non-tumorigenic epithelial cells were then loaded via electroporation, and the vesicles were evaluated to check the toxicity and uptake efficiency in both in vivo and in vitro experiments. The results proved that these exosome mimics can function as drug delivery nanocarriers targeting cancer cells [53].

On their part, liposomes are another type of nanovesicle that has been deeply studied, and they possess similar carrier usage to exosomes. However, they lack some properties that exosomes present, which are mostly conferred by the embedded membrane molecules in them and are not present in liposomes. Thus, liposomes present low blood circulation times, rendering them not as effective for therapeutic uses as they could be. Nonetheless, by changing the composition of liposomes to match the functions and behavior of an exosome, these limitations might be solved. An exosome-mimicking liposome was developed using dissolving lipid mixtures to acquire the desired composition, and it was then evaluated as a carrier for siRNA to target human lung carcinoma cells. The resulting vesicle was compared against different types of liposomes, including some contained in commercially available liposomal formulations. In general, they presented low cytotoxicity (around four times less than regular liposomes) and increased storage life, presenting little to no alteration after a 90-day storage. As for cellular uptake, the exosome-mimicking liposomes showed a threefold increase when compared to phosphatidylcholine liposomes specifically [54]. This last example is categorized inside the biohybrid approach, meaning all three strategies can be used to generate artificial exosomes with possible drug delivery applications. Further examples of each category can be found in Table 2.

## 3. Exosome Modification and Enhancement

**Figure 3:**
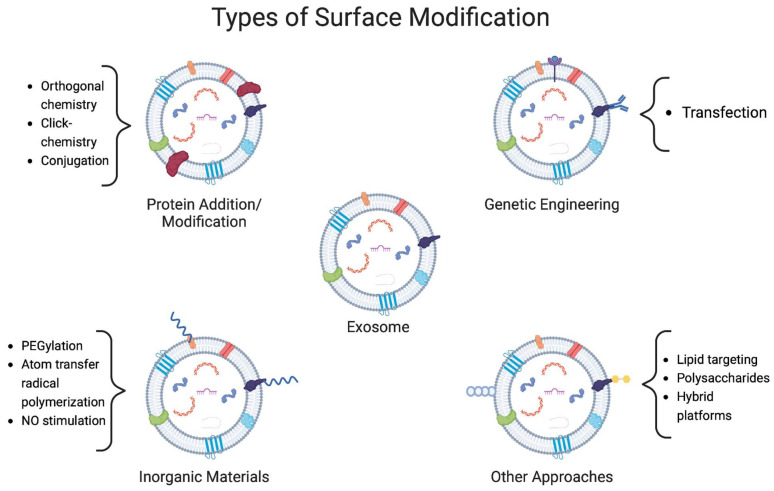
Representation of four different types of exosomal surface modifications. Strategies focus on the addition of different molecules to the membrane to confer the vesicles with protection and higher stability. Proteins and polymers are mainly used for this, however, some other materials such as polysaccharides have been used. A different strategy is the expression of certain molecules in the membrane, which is conducted by genetic engineering. Each strategy includes some examples of the methods used to achieve these modifications.

To optimize exosome drug delivery, several alternatives can be harnessed. However, one of the most common ones consists of making modifications outside the exosomal membrane. As mentioned, exosomes possess a lipid layer with embedded proteins, which can be modified to enhance its properties. Studies have shown that the lipid composition of exosomes is similar across different cell sources, making modifications in a specific group applicable to most of the cells [64]. Due to this lipidic-based composition, as well as their capacity for modification through the addition of new ligands, proteins and inorganic materials have opened the possibility for new treatments for various diseases [65]. This section will present different strategies that have been implemented to improve the therapeutic potential of exosomes via membrane-surface modification methods. It is important to note that some studies might combine different approaches to achieve even better results, mainly with genetic engineering and the addition of inorganic materials in the same formulation. A summary of the most recent strategies reported and employed for exosome-surface modification discussed in this section can be found in Table 3. Furthermore, in Figure 3, a more visual representation of these changes is schemed.

### 3.1. Protein Addition and Modification

The first strategy that has been used to provide different functionalities to the surface of exosomes was binding certain peptides or proteins that confer the vesicle a higher specificity or higher survivability in the organism while increasing its chance of reaching its target site and thus increasing its uptake. For instance, the cRGDyK peptide has been added to exosomes using orthogonal chemistry as a mean to pass the blood–brain barrier to assist the treatment of ischemic stroke. The trial was conducted using intravenous administration of curcumin-loaded vesicles in a mice model for anti-inflammatory purposes, showing an increase in the tropism of the exosomes in cerebral ischemic tissue and presenting a threefold improvement when compared to using naked exosomes [66].

Many studies also center on adding different ligands to the vesicles so that they increase their target capacity for certain cell types. For example, according to Lin et al., exosome-like vesicles were extracted from blood plasma and loaded with an anticancer agent (imperialine), and then the surface was modified by adding the integrin α3β1-binding octapeptide cNGQGEQc to target integrin α3β1, which is present in non-small cell lung cancer. This platform was capable of increasing the distribution and retention of the drug where tumors were present. Distribution and retention values were measured in different organs across 24 h, and at the end, the platform showed a concentration of more than 20 ng/mg in tumors, while the other two samples (i.e., imperialine and imperialine loaded exosomes) were less than 5 and 10 ng/mg, respectively [67].

The development of chimeric peptide-engineered exosomes for plasma-membrane- and nucleus-targeted photodynamic therapy has also been described. In this regard, it is expected that these modified vesicles will systematically destroy the plasma membrane and nucleus of tumor cells while leaving healthy cells untouched. The focus was to disrupt the integrity of the membrane, causing cell death while activating the production of reactive oxygen species inside the nucleus. The results provided interesting insights into this new possible therapy using radiation and nanovesicles, as it showed a good ability to target tumor cells and inhibit tumor growth [68].

A different approach to conjugating molecules to exosomes was reported by Smyth et al. In their study, they used copper-catalyzed azide alkyne cycloaddition—also known as click chemistry—to functionalize the exosomal membrane surface. Click chemistry refers to chemical reactions that have the following characteristics: selective, modular, high yielding and wide in scope [81]. In this previous example, the authors conjugated exosomes to a model azide-fluor 545, making use of these click chemistry reactions. The method was found to be highly applicable, as it may be used to bind the surface with small and large molecules as well as polymers without affecting neither the structure nor the function of the vesicles. It was also found that it might lead to an improvement in exosome tracking when contrast agents are present [69].

### 3.2. Genetic Engineering

The transfection technique is another strategy that has been used to provide exosomes with additional protein and ligand functionalization to increase their surface specificity. An advantage that this approach has over other modification forms is that it is possible to customize the exosome layer before harvesting. For instance, genetically modified K652 cells were used to produce exosomes with certain proteins (i.e., IL-15, IL-18 and 4-1 BBL), resulting in the activation of NK cells as well as promoting proliferation and offering a short-lived treatment targeting colorectal cancer cells. This treatment, however, can have a negative impact in the organism, as it can have the contrary effect by inhibiting the cytotoxicity of NK cells with a prolonged exposition of more than 48 h [70].

A more-complex demonstration of this process is the development of exosomes capable of targeting the tLyp-1 peptide, known to be specific in tumors and their connection network. This was conducted first by building a plasmid inside a model Escherichia coli, and then the constructed plasmid was transfected into HEK293T cells from where the modified exosomes were finally extracted and used to target cancer cells. The results showed a high transfection rate in the target cells, therefore proving that this modification method can be used for therapeutic applications [71].

Genetic engineering-based exosomes can also be used as a central structure for compound transport systems, meaning that they will present both peptides conferred by the mother modified cell as well as those attached for bonus characteristics. A system comprised of exosomes obtained from plasmid-transfected B16BL6 cells and expressing a streptavidin–lactadherin complex can be merged by biotinylating them with CpG DNA, thus making the modification complete. The cargo used in this system was endogenous tumor antigens, used to trigger an immune response and cascade into antitumoral effects [72].

Antibodies can also be expressed in the exosomal surface and form complex systems with other strategies by isolating them from cells in which these antibodies are present and coupling them with other inorganic materials. A33-presenting exosomes were isolated from LIM1215 cells and antibodies were bound with carboxyl superparamagnetic iron oxide nanoparticles. This molecule was used to target colon cancer cells, resulting in an increased uptake by the target cells, tumor antiproliferation effects and a rise in survivability in mice treated in in vivo experiments [73].

Even though there are many studies centering on cancer therapy applications, it is not the only possibility that has been explored; neural system applications are also an option. Rabies viral glycoprotein (RVG), a neuron-specific tumor, was expressed in an engineered exosome platform to carry the opioid receptor mu siRNA into the brain to aid in the treatment of morphine addiction relapse by causing downregulation in the receptor’s expression levels. This strategy showed a decrease in MOR mRNA of around 70% and MOR protein of around 25% compared to the control group [74].

### 3.3. Inorganic Materials

Using inorganic materials on the surface of exosomes can also prove to be highly beneficial for boosting certain elements for therapeutic processes. Polyethylene glycol (PEG) is a polymer that has been used to improve the general stability and increase the clearance time of therapeutic molecules from the organism. In the case of nanovesicles, macrophage-derived exosomes have been modified with anisamide and were then PEGylated (i.e., modified by covalently attaching PEG chains to their surface) to increase the cellular uptake and treat pulmonary metastases. The results seemed promising as this strategy proved to target only cancer-ridden cells and had positive effects in both in vitro and in vivo trials [75].

Another strategy using PEG-modified exosomes was explored by Shi et al., where positron emission tomography was used to monitor copper-64 radiolabeled PEG exosomes. The main use of this approach was to evaluate the imaging of the molecules as well as blood clearance and tumor uptake. PEG was shown to confer the exosomes with higher drug delivery quality against their non-PEGylated counterparts, providing new insights into the use of polymers for nanovesicle modification [76].

Different approaches can be taken for modifying the structure of the exosome membranes with polymers. For instance, exosomes have been functionalized with cholesterol-modified DNA tethers and complementary DNA block polymers; this process was conducted with various compatible polymers, resulting in a controlled and reversible functionalization process. The polymers were also grafted by using photomediated atom-transfer radical polymerization. The functionalized exosomes exhibited enhanced characteristics such as higher stability in storage and in the presence of proteolytic enzymes [82].

Alternatively, changing the outer structure of the exosomes can be achieved by modifying the environment in which they are produced. For example, mesenchymal stem cell exosomes were produced under the influence of an NO-releasing polymer, which resulted in an increase in the angiogenic-inducing effects found in the vesicles. Evidence suggests that this strategy may be used to produce specialized exosomes without the need for directly modifying either the cell or the nanovesicle [77].

### 3.4. Other Possible Exosome Modification Approaches

The lipid content on the surface of the exosomal membrane has also been targeted as a point of work, to improve its therapeutic effects. The development of a hybrid liposome-exosome was achieved by fusing both vesicles’ lipidic membranes via the freeze-thaw method. The result was an engineered exosome that could carry hydrophobic cargo in its membrane for regulation while carrying hydrophilic compounds inside for drug delivery systems [62].

Polysaccharides are another group that can be used to modify the functionality of these nanovesicles. Cationized pullulan, a fungal-sourced polysaccharide, was added via mixing due to the electrostatic interactions between the compounds. This combination had the objective of targeting injured liver cells through hepatocyte asialoglycoprotein receptors and optimizing the therapeutic ability of exosomes. This strategy seems useful as in vivo experiments reported that an accumulation of the modified exosomes in damaged liver tissue and promoted an anti-inflammatory response [78].

It has also been possible to create platforms to stray away from both the usually used genetic engineering and protein modification strategies. By developing the nanoassembly of DNA aptamers on the exosomal surface, a new possible method for modification was designed. This allowed for the use of molecular recognition between the DNA aptamers, offering a less-complex procedure with potential biomedical and analytical applications. However, it still needs to undergo clinical trials to see how this conjugate may affect the organism and whether it really is useful for therapeutic and analytical applications [79].

On their part, antibiotics, specifically ciprofloxacin, have been noted to have an effect on the surface of exosomes when the mother cells are exposed to the compound. The result was the release of nanovesicles with DNA and DNA-binding proteins. It was seen that this is the only case in which this process appears. However, this phenomenon has not been visualized for any application yet, so it is too early to say if this may help with the common uses designated for these vesicles or if it may develop new ones [80].

## 4. Conclusions

Exosomes and other extracellular vesicles have shown effectiveness when being used as drug delivery vehicles, but certain limitations have hampered their full application potential as well as their industrialization. There are various tools to rectify these problems. The scalability problem for mass production could be solved by extraction from by-products of the food industry. Furthermore, non-human animal fluids and plants could become a source that could greatly increase the cost-effectiveness of exosomes as therapeutic carriers, to enter different markets, but it is still too early to determine how viable these new sources might be. Nevertheless, among the sources presented in this review, plant-based and animal-based extracellular nanovesicles seem the more viable options both for economic and industrial production. However, there is still scarce information on their potential applications and procurement. Regarding the therapeutic potential enhancement for these vesicles, genetic engineering and the use of inorganic nanomaterials have shown promising results. The obtention of small extracellular vesicles from alternative sources is a novel area of research in which undoubtedly new limitations will appear. Information on the subject is still scarce, so as of right now, it is hard to say if production from these sources will represent even more challenges. However, because of the abundance of these materials and/or their ease of modification, it is expected that this could compensate the difficulties that may arise from the use of these alternative sources. The next step will be to observe the evaluation of these new platforms in clinical studies as well as their performance. A lot of refinement in many areas still needs to be conducted, including the extraction, standardization, and functionalization of the extracellular vesicles, before more consistent results are achieved and new clinical applications are discovered. There is potential for opening new avenues and uncovering treatments for all kinds of diseases, but all these limitations must be tackled first. With new advancements in strategies to improve the pharmacokinetic and pharmacodynamic characteristics of exosomes and other extracellular nanovesicles, better and customized therapies might be achieved. However, the most effective strategies appear to combine various modifications to ensure optimal specificity and functionality.

## Figures and Tables

**Table 1:** Types of clinical applications for nanovesicles.

Source	Type	Application	Strategy	Reference
Mesenchymal stromal cells	Drug delivery system	Cancer therapy	Doxorubicin loaded exosomes	[11]
Macrophages	Drug delivery system	Parkinson’s disease therapy	Catalase loaded exosomes	[12]
Mouse bone marrow	Drug delivery system	Cerebral ischemia therapy	Various strategies	[13]
Human breast carcinoma cell line	Biomarker	Diagnosis and prognosis of breast cancer	Microfluidic chip immunocapture	[14]
Mouse blood serum	Biomarker	Alzheimer’s disease early-stage detection	Detection of Aβ42 oligomers in exosomes	[15]
Urinary fluid	Biomarker	Prostate cancer detection	Sex steroid exosome analysis	[16]
Lung cells	Vaccine	COVID-19 inhalable vaccine	Conjugation with viral receptor-binding domain	[17]
MRC-5 infected cells	Vaccine	Rabies virus infection	Delivery of inducible miR-423-5p	[18]
Mouse dendritic cells	Vaccine	Leishmania major immunity	Antigen loaded dendritic cells exosomes	[19]

**Table 2:** Different design approaches for synthetic exosomes construction.

Method	Base Material	Strategy	Application	Reference
Serial Extrusion	MDA-MB-231 cells	Top-down	Clinical uses	[55]
	Human mesenchymal stem cells	Top-down	Bone regeneration	[56]
	Hepatocytes	Top-down	Liver regeneration	[57]
Protein addition	Liposomes and lipids	Bottom-up	Lung cancer therapy	[58]
Microfluidics	Lipid mixture miRNA	Bottom-up	Gene expression	[59]
Incubation and centrifugation	Liposomes and exosome components	Bottom-up	SiRNA delivery	[60]
Membrane fusion	EVs and lipids	Biohybrid	Gene delivery carriers	[61]
	Liposomes and exosomes	Biohybrid	Drug delivery systems	[62]
Simple incubation	Liposomes and exosomes	Biohybrid	CRISPR/Cas9 delivery	[63]

**Table 3:** Summary of the different types of exosome modifications.

Modification	Type	Application	Reference
cRGDyK peptide	Protein addition	Pass the blood–brain barrier Treatment of ischemic stroke	[66]
Integrin α3β1-binding octapeptide cNGQGEQc	Protein addition	Target non-small lung cancer Increase distribution and retention in tumor cells	[67]
Chimeric peptide	Protein addition	Targeted photodynamic therapy	[68]
Click chemistry	Protein addition	Novel approach for ligand addition Therapeutic applications	[69]
IL-15, IL-18 and 4-1 BBL protein production	Genetic engineering	Activation of NK cells Treatment of colorectal cancer	[70]
tLyp-1 peptide	Genetic engineering	Target tumor cells and connections Therapeutic applications	[71]
Streptavidin-lactadherin and CpG DNA	Genetic engineering	Trigger autoimmune response Antitumoral effects	[72]
Antibody A33 and carboxyl superparamagnetic iron oxide nanoparticles	Genetic engineering/Inorganic materials	Target colon cancer cells Antiproliferation effects	[73]
Rabies viral glycoprotein	Genetic engineering	Morphine addition relapse treatment Receptor downregulation	[74]
Polyethylene glycol and Anisamide	Inorganic materials/Genetic engineering	Increase the cellular uptake Treat pulmonary metastases	[75]
Copper-46 PEG	Inorganic materials	Improved drug delivery	[76]
NO affected exosomes	Inorganic materials	Angiogenic inducing effects Therapeutic applications	[77]
Cationized pullulan	Other approaches	Target injured liver cells	[78]
DNA nanoassembly	Other approaches	Still in trials to see functionality Classic and new applications	[79]
Ciprofloxacin influenced DNA expressing vesicles	Other approaches	Unknown, may complement or develop new applications	[80]

## Data Availability

Not applicable.
